# Fibrinogen glycosylation and glycation: molecular insights into thrombosis and vascular disease

**DOI:** 10.3389/fmolb.2025.1680332

**Published:** 2025-09-24

**Authors:** Serena Borghi, Francesca Nencini, Elvira Giurranna, Ilenia Barbaro, Niccolò Taddei, Claudia Fiorillo, Matteo Becatti

**Affiliations:** Department of Experimental and Clinical Biomedical Sciences “Mario Serio”, University of Firenze, Firenze, Italy

**Keywords:** thrombosis, fibrinogen, glycosylation, glycation, cardiovascular diseases, post-translational modifications

## Abstract

Fibrinogen, a key protein in blood coagulation, undergoes two distinct post-translational modifications (PTMs): glycosylation and glycation. Glycosylation is an enzymatic, tightly regulated process, whereas glycation occurs non-enzymatically under hyperglycemic conditions. Emerging evidence highlights the role of these modifications in cardiovascular risk. This review provides a comprehensive overview of how fibrinogen glycosylation and glycation contribute to altered haemostatic profiles and increased cardiovascular risk. Evidence is presented from inherited fibrinogen disorders, liver disease, diabetes, and chronic conditions such as end-stage renal disease. Additionally, the potential use of glycosylation and glycation patterns as diagnostic or prognostic biomarkers in cardiovascular disease is discussed. Overall, changes in fibrinogen’s glycosylation and glycation profiles may serve as important markers for cardiovascular risk assessment in many diseases, offering insights into the molecular mechanisms underlying these conditions.

## 1 Introduction

Glycosylation and glycation both involve the modification of proteins by carbohydrates; however, their molecular mechanisms and their functions in physiology and pathology are very different. Protein glycosylation is an enzymatic, highly regulated process integrated into protein biosynthesis, while glycation is a non-enzymatic, random process triggered by hyperglycemia, occurring in both extracellular or intracellular environments (Uceda et al., 2024; Schjoldager et al., 2020; Taniguchi et al., 2016). Recently, the relevance of protein carbohydrate moieties as a novel biomarker in cardiovascular risk has been proposed (Chatham and Patel, 2024). Fibrinogen is a central protein in blood coagulation, a soluble macromolecule that forms an insoluble fibrin clot by the action of thrombin, which is activated by a cascade of enzymatic reactions (Kattula et al., 2017). The extensive implications of post translational modifications (PTMs) on fibrinogen are well-known, and a deeper understanding of these modifications is crucial for advancing our knowledge in haemostasis and thrombosis (Becatti et al., 2014; Becatti et al., 2020; Gitto et al., 2024; Nencini et al., 2024; Nencini et al., 2025a). Clot formation, stability, and resistance to degradation, can be greatly influenced by PTMs, such as glycosylation and glycation, thereby increasing a prothrombotic phenotype (Nencini et al., 2024; Giurranna et al., 2025; Nencini et al., 2025b). Building on this understanding, the present review explores the pathological and clinical relevance of fibrinogen glycosylation/glycation in cardiovascular risk, with a focus on how these modifications influence clot properties and the protein’s immunogenicity.

## 2 Protein glycosylation vs. protein glycation

Protein glycosylation, the covalent attachment of carbohydrates to specific amino acids, is one of the most abundant co-translational and post-translational modifications in eukaryotic cells. This modification occurs in various cellular compartments, including the endoplasmic reticulum (ER), Golgi apparatus, nucleus, cytoplasm, and mitochondria (Schjoldager et al., 2020). Glycosylation can be mainly classified based on the site and nature of the carbohydrate attachment: N-glycosylation, where carbohydrates are attached to the amide side chain of asparagine (Asn), and O-glycosylation, in which glycans are attached to the hydroxyl group of side chains of serine (Ser) or threonine (Thr). Residues that undergo glycosylation are within a consensus sequence (Chatham and Patel, 2024; Kobata, 1992; Hevér et al., 2019).

N-glycosylation is a co-translational modification, that begins in the ER and involves the attachment of a conserved oligosaccharide core (2 N-acetylglucosamines and 3 mannose residues). This process requires numerous glycosidases and glycosyltransferases, resulting in a mixture of glycosylated protein variants. In contrast, O-glycosylation is strictly post-translational. It begins with the addition of GlcNAc to the hydroxyl group of Ser or Thr, followed by elongation into various core structures that form linear or branched polysaccharide chains. O-linked glycans are classified by their initiating monosaccharides (Schjoldager et al., 2020; Hevér et al., 2019). Terminal sialic acid and fucose residues diversify glycan structures and confer functions such as protein stabilization, modulation of interactions, and ion transport. Loss of sialic acid generates asialylated glycans, which are rapidly cleared from circulation by hepatic receptors (Chatham and Patel, 2024). Protein N- or O-glycosylation alters structure and function, profoundly affecting biological activity and processes such as receptor interaction, immune response, secretion, and transport (Schjoldager et al., 2020; Chatham and Patel, 2024; Rudd et al., 2001; Czuba et al., 2018); it also can affect protein properties such as aggregation, solubility, stability, and folding (Ma et al., 2024; Jayaprakash and Surolia, 2017). Given their biological relevance, alterations in protein glycosylation are associated with the pathogenesis of many diseases (Spiro, 2002), such as cancer (Huang et al., 2021), infections (Pandey et al., 2022), or autoimmune disorders (Lu et al., 2023).

Glycation is a non-enzymatic reaction that starts with the interaction between reducing sugars, or their autoxidation products, and the amino groups of proteins. This process is most associated with hyperglycemic conditions, such as diabetes mellitus (DM), and aging. Unlike other PTMs, glycation lacks a defined physiological function. Nevertheless, it has a clinical significance as indicated by the detrimental consequences that it causes (Uceda et al., 2024; Taniguchi et al., 2016; Shin et al., 2023). Glycation begins with Schiff base formation between reducing sugars and protein amino groups, followed by Amadori rearrangement to stable products that ultimately give rise to advanced glycation end-products (AGEs), a heterogeneous and harmful class of compounds (Shin et al., 2023; Vistoli et al., 2013). Similarly, glycation of nucleophilic groups of other long-lived biomacromolecules, such as amino phospholipids of the outer leaflet of cell membranes, leads to the advanced lipoxidation end products (ALEs) (Vistoli et al., 2013).

AGEs and ALEs exert their damaging activity through the loss of function of the target proteins, covalent modification of enzymes and receptors, as well as immunogenic effects (Vistoli et al., 2013; Kurien et al., 2006; Shamsi et al., 2016). Glycolysis generates reactive dicarbonyls like methylglyoxal (MGO) and glyoxal (GO), whose reactivities far exceed glucose (by 200–50,000-fold) despite their lower concentrations (Shin et al., 2023). MGO is a main contributor of dicarbonyl stress, which manifests as oxidative stress, inflammation, aging, and hyperglycemia (Wang et al., 2022; Wang et al., 2024; Todoriki et al., 2022). Additionally, MGO is present in nearly all foods, with its levels increasing during heating, fermentation, and extended storage (Vistoli et al., 2013). Dietary intake of glycation compounds and disease, including allergy, diabetes, cardiovascular and renal disease, gut gastrotoxicity, cognitive impairment, and cancer, has been extensively reviewed (Hellwig et al., 2024).

Glycation is countered by defense systems: the glyoxalase pathway detoxifies MGO/GO, while proteasomal degradation and autophagy remove structurally damaged glycated proteins (Uceda et al., 2024; Shin et al., 2023; Hellwig et al., 2024).

### 2.1 Protein glycosylation and glycation: novel biomarkers into cardiovascular disease

In 2010, the American Heart Association (AHA) introduced the Life’s Simple 7 model to define cardiovascular health, encompassing seven metrics: four health behaviors (diet, physical activity, smoking, BMI) and three clinical factors (cholesterol, blood pressure, blood glucose) (Martin et al., 2025).

Despite the general decline in age-adjusted cardiovascular disease (CVD) death rates, the absolute number of CVD deaths continues to rise. Globally, CVD remains the leading cause of death, accounting for approximately 18 million deaths in 2017, underscoring the urgent need for early detection biomarkers (Dashti et al., 2021).

The potential of glycans as biomarkers in CVD originates from the systemic pro-inflammatory response, which triggers hepatic release of acute-phase proteins (Chatham and Patel, 2024; Ballout and Remaley, 2020; Connelly et al., 2016) including fibrinogen, C-reactive protein (CRP), haptoglobin, serum amyloid A (SAA), all of which are primarily N-linked glycoproteins. As inflammation progresses, N-glycosylation patterns become more complex through residue extensions, increased branching, or loss of sialic acid/galactose (Connelly et al., 2016; Otvos et al., 2015; Jamieson et al., 1983).

To quantify this glycan-based inflammatory response, the GlycA test was developed. This test quantifies glycan-driven inflammation by measuring NMR signals from N-acetylglucosamine residues on plasma proteins (Ballout and Remaley, 2020; Otvos et al., 2015). Higher baseline GlycA levels predict incident CVD events with a risk magnitude comparable to high-sensitivity CRP (hsCRP) (Akinkuolie et al., 2014). In the Multi-Ethnic Study of Atherosclerosis (MESA), GlycA was positively associated with all-cause mortality, CVD, cancer, chronic inflammatory-related disease, hospitalization, and death, independently of hsCRP, IL-6, and D-dimer (Dupr et al., 2016). Moreover, GlycA and hsCRP were respectively linked to myocardial infarction and ischemic stroke in a multi-ethnic pooled cohort (Riggs et al., 2022), and elevated GlycA was further associated with impaired HDL function and metabolism (Riggs et al., 2019). Moreover, elevated circulating levels of glycoprotein N-acetyl methyl groups have been shown to predict long-term risk of all-cause, cardiovascular, and cancer mortality in initially healthy individuals (Lawler et al., 2016).

Alterations in immunoglobulin G (IgG) N-glycosylation patterns have emerged as promising predictors of CVD events (Dashti et al., 2021; Hoshi et al., 2024). In two nested case-control studies, six distinct IgG N-glycan structures were significantly associated with incident CVD (Hoshi et al., 2024). Menni et al. further identified four IgG glycan traits linked to carotid plaque formation, independent of conventional risk factors (Menni et al., 2018). In coronary artery disease (CAD), sex-stratified analysis showed that sialylated N-glycan structures were negatively associated with CAD in women (Radovani et al., 2023).

Glycans and sialylation regulators are emerging as potential biomarkers and therapeutic targets for atherosclerosis, owing to their abundant presence on the vascular endothelium and on circulating lipoproteins implicated in plaque formation (Wattchow et al., 2025; Pu and Yu, 2014).

Sialylation plays a key role in atherosclerosis regulation. Desialylation of LDL by human neuraminidases represents a novel pro-atherogenic pathway (Demina et al., 2021). ST6GAL1-mediated sialylation regulates adhesion molecules and chemokine receptors, influencing monocyte recruitment in early atherogenesis (Dashti et al., 2021). In a ApoE−/− mice fed a high-fat diet, vascular ST6GAL1 expression decreased during atherosclerosis progression and was restored after regression with rosuvastatin treatment (Zhang et al., 2018).

AGEs and glycosylation contribute to cardiovascular remodeling in metabolic disorders such as obesity, diabetes, and metabolic syndrome, through direct effects (elevated AGEs, ROS, inflammation) and indirect mechanisms via comorbidities including atherosclerosis, MI, heart failure, and atrial fibrillation (AF) (Dozio et al., 2021). Circulating and tissue AGEs correlated with metabolic imbalance and chronic CVD progression (Yubero-Serrano and Pérez-Martínez, 2020), with both dietary and endogenous AGEs acting as cardiometabolic risk factors (Luévano-Contreras et al., 2017). AGEs promote endothelial dysfunction and vascular stiffening (Dozio et al., 2021; Yubero-Serrano and Pérez-Martínez, 2020), and have been associated with arterial stiffness independent of glycemia (Birukov et al., 2021). Their synthesis is accelerated under oxidative stress and inflammation, where ROS and lipid peroxidation products (GO, MGO) drive the formation of AGEs (Dozio et al., 2021; Shen et al., 2020).

Furthermore, glycation products, such as glycated hemoglobin (HbA1c) and glycated albumin (GA), are widely used in clinical practice as biomarkers of glucose homeostasis in DM and are potential prognostic factors for DM-associated diseases (Dozio et al., 2021). However, data from 73 prospective studies indicate that while HbA1c provides modest improvements in CVD risk prediction, its added value is limited (Di Angelantonio et al., 2014). To address individual variability in HbA1c response, the hemoglobin glycation index (HGI) has been introduced as a corrective measure for personal glycation bias. In patients with diabetes and coronary artery disease, HGI displayed a U-shaped association with major adverse cardiac events, with lower HGI values linked to an increased risk of cardiovascular death over 3 years (Lin et al., 2024). The REACTION cohort study explored the same relationship in a period of 5 years, rediscovering the same U-shaped correlation between the HGI values and the risk of 5-year MACE (Wang et al., 2023).

GA is gaining interest as a more accurate short-term indicator of glycemic control (Rooney et al., 2022). A meta-analysis of cohort studies showed that elevated GA levels are associated with higher risk of CVD and mortality, both in patients with and without dialysis (Zhao et al., 2023).

Collectively, these findings highlight the potential of protein glycation and glycosylation profiles as emerging biomarkers for cardiovascular risk assessment. A deeper understanding of the underlying mechanisms may also foster the development of novel therapeutic strategies aimed at delaying or preventing related complications.

## 3 Fibrinogen: synthesis, structure and inherited disorders

Fibrinogen is a complex fibrous hexameric glycoprotein with a molecular weight of 340-kDa, consisting of two copies of three distinct polypeptide chains (2Aα, 2Bβ, and 2γ), held together by 29 disulfide bonds in a dimer with bilateral symmetry. The predominant Aα chain is composed of 610 amino acids, the Bβ chain contains 461 amino acids, and the γ 410 amino acids. Structurally, the fibrinogen molecule is about 45 nm in length, with globular domains at each end, and connected in the middle by α-helical coiled-coil rods. Molecular masses of Aα, Bβ, and ϒ chains are 66.5, 52, and 46.5 kDa, respectively. The posttranslational addition of asparagine-linked carbohydrate to the Bβ and ϒ chains brings the total molecular mass to about 340 kDa.

Fibrinogen assembly occurs in a stepwise process: initial formation of Aα–γ and Bβ–γ complexes, followed by their combination into Aα/Bβ/γ half-molecules, and ultimately into the complete hexameric form, (Aα/Bβ/γ)_2_. All six N-terminal regions converge at the central E domain of the molecule, while the C-termini of the Bβ and γ chains extend outward into distal D domains. The Aα chains’ C-termini are globular and situated near the central E domain of fibrinogen, where they interact intramolecularly. This complex assembly process takes place within the endoplasmic reticulum of the cell (Kattula et al., 2017; Weisel, 2005; Güven and Can, 2024).

Fibrinogen synthesis occurs primarily in hepatocytes, where fibrinogen mRNAs are spliced and translated into polypeptide chains. These chains then undergo PTMs, including the attachment of oligosaccharide units via N-glycosidic bonds. This glycosylation is essential for proper fibrin polymerization and clot architecture. Then, fibrinogen is secreted into the blood, where it circulates at concentrations ranging from 200 to 400 mg/dL (Dobson et al., 2024; Wolberg, 2023). After translation, each polypeptide chain is independently translocated into the lumen of the ER, and then the interaction of the chains with chaperone proteins leads to the assembly and folding processes of the protein. Quality control mechanisms guarantee proper assembly of fibrinogen and its secretion out of the lumen, while unassembled forms are degraded (Dobson et al., 2024; Weisel and Litvinov, 2017).

Fibrinogen is encoded by three closely linked genes (FGA, FGB, and FGG), each specifying the primary structure of one of its three polypeptide chains, Aα, Bβ, and γ, respectively. Mechanisms that regulate fibrinogen gene expression are still largely undetermined (Wolberg, 2023). These genes are clustered on chromosome 4, and they translate into nascent polypeptides of pre-pro-chain. Interestingly, overexpression of any one of these genes appears to upregulate the expression of the others, suggesting coordinated regulation.

While the exact regulatory mechanisms controlling fibrinogen gene expression remain largely unclear, alternative splicing events are known to contribute to fibrinogen diversity. For example, a longer Aα chain (αE-chain) variant is produced in about 1%–2% of fibrinogen molecules. The FGG transcript also undergoes alternative splicing with a major γ chain mRNA from 10 exons, and a minor γ′ chain mRNA (about 10%) (Fish and Neerman-Arbez, 2012). The γ′ chain variant is of particular interest, as its presence has been linked to alterations in fibrin structure and is considered relevant in the context of thrombosis (Duval and Ariëns, 2017).

Variants in the fibrinogen genes are directly associated with inherited fibrinogen disorders and can be classified into quantitative and qualitative anomalies, as extensively reviewed (De Moerloose et al., 2013; Neerman-Arbez et al., 2016; Neerman-Arbez, 2006). Briefly, congenital fibrinogen disorders (CFDs) include dysfibrinogenemia, characterised by normal circulating levels of fibrinogen with abnormal function, and hypofibrinogenemia/afibrinogenemia, which are characterised by reduced (<1.5 g L^-1^) or absent fibrinogen levels in the blood, respectively. A combination of low concentration and dysfunctional fibrinogen is defined as hypodysfibrinogenemia (Fish and Neerman-Arbez, 2012; Neerman-Arbez, 2006). Dysfibrinogenemia and hypodysfibrinogenemia are typically autosomal dominant disorders caused by heterozygous mutation (rarely homozygous) or compound heterozygous mutation in one of the fibrinogen genes. While many patients with dysfibrinogenemia are asymptomatic, some may experience bleeding, thromboembolic events, or both (Neerman-Arbez et al., 2016). Hypofibrinogenemia and afibrinogenemia are most often caused by heterozygous mutation, but also by homozygous or compound heterozygous mutation, in one of the FGA, FGB, and FGG genes. Afibrinogenemia, characterized by the complete absence of fibrinogen, is the most severe form of CFDs. Most mutations responsible for these forms are null mutations, which result in complete lack of protein production, or are missense or late-truncating nonsense mutations that produce abnormal chains. These defective chains are synthesized but retained within the cell due to impaired assembly or secretion of the fibrinogen complex (de Moerloose et al., 2013; Neerman-Arbez et al., 2016).

Afibrinogenemia is associated with mild-to-severe bleeding, whereas hypofibrinogenemic patients often exhibit few symptoms or remain asymptomatic (de Moerloose et al., 2013; Vu and Neerman-Arbez, 2007). In populations where consanguineous marriages are common, the incidence of afibrinogenemia, as for other autosomal recessive coagulation disorders, is increased (Neerman-Arbez et al., 2016). An interesting subset of patients with hypofibrinogenemia has accompanied liver disease characterized by endoplasmic reticulum fibrinogen-positive liver inclusions. This rare condition is known as hereditary hypofibrinogenemia with hepatic storage (HHHS). To date, only eight mutations affecting fibrinogen γ chain have been reported to cause HHHS (Casini et al., 2018; Asselta et al., 2020).

Clinical management of patients with CFDs is challenging. Replacement therapy is the mainstay treatment for bleeding episodes and varies by geographic region. Depending on available resources, patients may receive fresh frozen plasma, cryoprecipitate, or fibrinogen concentrates (De Moerloose et al., 2013; Casini et al., 2018).

## 4 Fibrinogen post translational modifications by carbohydrates

Fibrinogen’s structural and functional variability depends on congenital disorders, genetic polymorphisms, alternative mRNA splicing, and a wide range of PTMs (Nencini et al., 2024). PTMs increase protein complexity, affecting structure and function (Becatti et al., 2025; Fini et al., 2024; Bettiol et al., 2023; Emmi et al., 2021; Whittaker et al., 2017; Dinu et al., 2018). Numerous studies have emphasized the potential role of post-translationally altered fibrinogen with the formation of prothrombotic fibrin clots (Becatti et al., 2014; Fini et al., 2024; Bettiol et al., 2023; Becatti et al., 2016; Lipinski and Pretorius, 2012; Vadseth et al., 2004; Damiana et al., 2020; Cellai et al., 2013).

**FIGURE 1:**
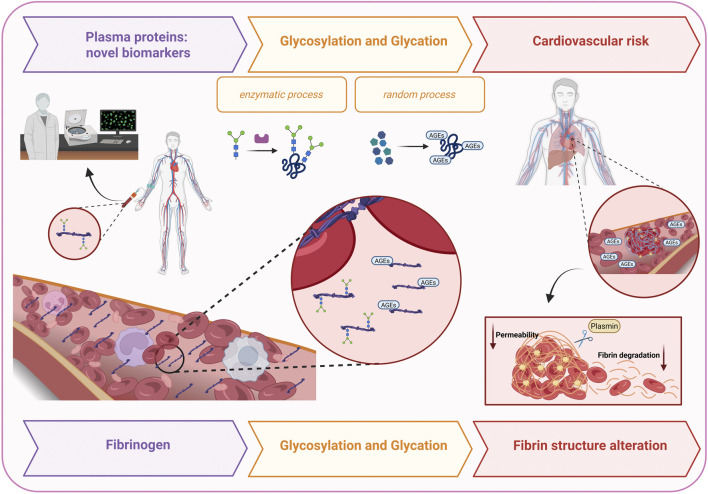
This figure illustrates the proposed mechanism by which glycosylation- and glycation-induced alterations in fibrinogen structure and function contribute to cardiovascular risk. Fibrinogen glycosylation profiles are suggested as potential biomarkers in several diseases, including atrial fibrillation, COVID-19, chronic thromboembolic pulmonary hypertension, and diabetes. While glycosylation is an enzymatic covalent attachment of carbohydrates to proteins, glycation is a non-enzymatic reaction with reducing sugars. These modifications promote α-helix to β-sheet transitions, amyloid-like aggregate formation, and altered fibrin networks, leading to denser, less porous clots resistant to plasmin-mediated lysis.

We review the current literature on human fibrinogen glycosylation and glycation, focusing on specific modification sites, the biochemical pathways involved, and their impact on fibrin clot properties, to elucidate potential correlations with cardiovascular risk (Table 1). Figure 1 illustrates the proposed mechanism by which glycosylation- and glycation-induced structural and functional alterations of fibrinogen contribute to cardiovascular disease. Moreover, we propose fibrinogen glycosylation profiles as potential biomarkers across several pathological conditions.

**TABLE 1:** Glycosylation and glycation affect fibrin (ogen) structure and function.

			Polymerization			Structure	Clot analysis					
	Ref.	Method	Lag phase	Max Abs	Vmax	Fibrinogen structural analysis	Aggregation	Fiber diameter	Stiffness	Permeability	Density	Fibrin lysis
Effect of glycosylation												
Dysfibrinogenemia and gain-of-glycosylation mutation	Marchi et al. (2004)	F. Lima extra N-glyc. Aα Asn139	**=**	**−**	**−**			**−**	**+**	**−**	**+**	
	Woodhead et al. (1996)	F. Caracas II Aα Ser434 to N-glyc Asn						**−**	**=**	**+**		
	Sugo et al. (1999)	F. Niigata BβAsn160 to Ser sub, extra glyc. Bβ Asn158	**+**	**−**	**−**			**=**		**=**	**+**	
	Ridgway et al. (1997)	F. Kaiserslautern γ380Lys to N-glyc Asn	**+**	**−**	**−**			**−**				**=**
	Yamazumi et al. (1989)	F. Asahi γ310Met to Thr sub, extra N-glyc. Asn308	**+**	**−**	**−**							
Increased glycans content	Baralić et al. (2023)	Fibrinogen from healthy persons age range 21–83		**=**	**−**	increase in tryptophan signal		**=**		**=**		
	Martinez et al. (1983)	Dysfibrinogenemia associated with liver disease (fibrinogen from 3 patients)	+	−	−							
	Martinez et al. (1978)	Dysfibrinogenemia associated with liver disease (fibrinogen from 12 patients)			**−**							
Increased glycans content	Maghzal et al. (2005)	Fibrinogen from 12 patients on fibrate therapy		**−**	**−**							
	Hansen and Schousboe (1984)	F Copenhagen II	**+**	**−**	**−**							
Decreased glycosylation	Langer et al. (1988)	Fibrinogen + PNG-Asn amidase		**+**	**+**			**+**		**+**		
	Maghzal et al. (2005)	Fibrinogen from 11 pregnant women	**−**		**+**							
Changing in glycosylation pattern	Gligorijević et al. (2018a)	Fibrinogen from 20 cirrhotic patients				Altered CD spectra and reduction in fluorescence spectra						
	Nagel et al. (2018)	Fibrinogen from HCC and/or LC patients and healthy donors				Decreased O-glyc in Aα-chain in LC, increased sialyl/fuc of N-type glyc in Bβ- γ-chain in LC/HCC						
Effect of glycation												
Fibrinogen + glucose/ribose	Robi et al. (1991)	Fibrinogen +500 mM glucose at 37 °C for 48 h	**+**	**+**	**+**							
	Brownlee et al. (1983)	Fibrinogen +50- or 500-mM glucose/G6P 23 °C for 21 days										**−**
Fibrinogen + glucose/ribose	Khanam et al. (2021)	Fibrinogen +10–25- or 50 mM D-ribose 37 °C for 7 days				structural alterations in UV-vis fluorescence, CD spectra, SEM FTIR spectra	**+** (aggregation)					
	Svensson et al. (2012)	Fibrinogen +20- or 100-mM glucose 37 °C for 24 h, 5 or 7 days				GlcK-133 and GlcK-75 within the plasmin sensitive region						
	Norton et al. (2017)	Fibrinogen + 5–10-, 25-, or 50-mM glucose 37 °C for 2 or 4 days					Glycated fibrin structures are more aggregated and anisotropic					
	Mirmiranpouret al. (2012)	Fibrinogen + 0–400 mM glucose 37 °C for 4 months				Increased fluorescence intensity, reduced α-helix, increased β-sheet						
	Hood et al. (2018)	Fibrinogen + 6-or 10-mM glucose for 48 h					decreased fibrin fiber overlapping				**+**	**−**
	Krantz et al. (1987)	Fibrinogen +20-or 50-mMglucose for more than 8 days		**+**			**+**					**−**
Fibrinogen + glucose/ribose	Mirshahi et al. (1987)	Fibrinogen +100 mM glucose, different timing										**−**
	Luzak et al. (2020)	Fibrinogen +30 mM glucose 37 °C for 4 days		**−**	**−**							
Fibrinogen + MGO	Perween et al. (2022)	Fibrinogen +7.5-mM MGO 37 °C for 0, 7, or 14 days				Reduced α-helix content and altered FTIR spectra	**+** (aggregation)					
	Rehman et al. (2020)	Fibrinogen + MGO				α-helix shift to β-sheet, altered FTIR spectra	**+** (aggregation)					
	Perween et al. (2019)	Fibrinogen +1, 5, 7.5, 10 or 15-mM MGO 37 °C for 7 days				Reduced intrinsic fluorescence and α-helix content increased β-sheet and carbonyl content	**+** (aggregation)					
	Alouffi et al. (2022a)	Fibrinogen +25, 50-mM GO 37 °C for 1–15 days				Reduced intrinsic fluorescence, altered FTIR and CD spectra, increased carbonyl content	**+** (aggregation)					
Type II diabetic subjects	Robi et al. (1991)	Fibrinogen from 11 diabetic and 18 non-diabetic subjects	**+**	**+**	**+**							
	Dunn et al. (2005)	Fibrinogen purified from 150 diabetic patients and 50 control subjects	**−**	**+**				**+**		**−**	**+**	
	Li et al. (2016)	Plasma from 5 diabetic patients, before and after blood glucose control and 4 control subjects						**−**			**+**	
	Luzak et al. (2020)	Plasma from 27 diabetic patients and 22 control subjects		**=**	**−**							
	Dunn et al. (2006)	Fibrinogen purified from 150 diabetic patients and 50 control subjects										−
	Pieters et al. (2008)	Fibrinogen purified from 20 diabetic patients and 18 control subjects			**+**			**=**	**=**	**−**		**−**
	Pieters et al. (2006)	Fibrinogen purified from 20 diabetic patients and 20 control subjects	**=**	**=**	**=**					**=**	**=**	
Type I diabetic subjects	Jörneskog et al. (2003)	Fibrinogen purified from 28 type I diabetic patients, before and after 4–6 months with CSII						**−**		**−**		

This table summarizes the methodologies and main findings of the reviewed studies, with outcomes centered on fibrin polymerization, fibrinogen structural alterations, and clot properties. It is organized into two sections: (1) effects of fibrinogen glycosylation and (2) effects of fibrinogen glycation. Symbols denote the direction of functional or structural changes: “=” no change, “+” increase, and “−” decrease.”

Asn: Asparagine; CD: circular dichroism; CSII: continuous subcutaneous insulin infusion; F: fibrinogen; FTIR: fourier transform infrared; GO: glyoxal; G6P: Glucose-6-Phosphate; HCC: hepatocellular carcinoma; LC: liver cirrhosis; Lys: Lysine; Met: Methionine; MGO: methylglyoxal; N-glyc: N-glycosylation; SEM: scanning electron microscopy; Ser: Serine; Thr: Threonine.

### 4.1 Fibrinogen post translational modifications: glycosylation

The N-glycosylation profile of human fibrinogen has been described by Adamczyk et al. to determine its contribution to distinguishing serum and plasma N-glycome. Using a combination of complementary chromatographic and mass spectrometry-based techniques, the study identified biantennary digalactosylated monosialylated (A2G2S1) and disialylated (A2G2S2) glycans as the most prevalent forms, comprising approximately 53.0% and 32.6% of fibrinogen N-glycans, respectively (Adamczyk et al., 2013). Normally, the α-chain of fibrinogen is not N-glycosylated, even though it has two potential N-glycosylation sites at Asn453 and Asn686 (Weisel, 2005; Clerc et al., 2016). Interestingly, deglycosylation of fibrinogen accelerates the polymerization rate and increases lateral aggregation of fibrin fibers. Constitutive glycosylation influences fibrinogen behaviour by exerting a repulsive force that maintains solubility and restricts fibrin assembly, thereby impacting clot architecture (Langer et al., 1988).

However, several mutations introduce novel glycosylation consensus sequences, resulting in added molecular mass and altered protein charge. The location of extra oligosaccharides in some abnormal fibrinogen variants may provide insight into their effects on fibrin polymerization (Marchi et al., 2004; Woodhead et al., 1996; Sugo et al., 1999). It has been described gain-of-glycosylation mutation on the Aα chain, Aα Arg141 to Ser mutation (Marchi et al., 2004) and Aα Ser434 to Asn mutation (Woodhead et al., 1996), which both create new N-glycosylated sites. The first one, fibrinogen Lima, is an abnormal fibrinogen resulting in an extra N-glycosylation at Aα Asn139, which seems to be responsible for the impairment of fibrin polymerization. Clot properties were examined in both homozygous and heterozygous forms, revealing that homozygous clots consist of thinner fibers with reduced permeability and altered rheological characteristics. The extra carbohydrate moiety impairs the protofibril lateral association process, giving rise to thinner, more curved fibers, with the structural anomalies being most pronounced in the homozygous clots (Marchi et al., 2004). Fibrinogen Caracas II is an abnormal fibrinogen involving the mutation of Aα Ser434 to N-glycosylated Asn, but this kind of dysfibrinogenaemia is asymptomatic. Clots formed show large pores bounded by thin fiber networks, with normal viscoelastic properties (Woodhead et al., 1996).

Another inherited dysfibrinogenemia (the Niigata variant), affecting the Bβ chain with a Bβ Asn160 to Ser substitution is associated with an extra glycosylation at Bβ Asn158. While fibrin polymerization and clot stiffness remain largely unaffected, the lateral aggregation of protofibrils may be subtly impaired (Sugo et al., 1999). Recently, Asselta et al. identified a novel Bβ Asp185Asn mutation in a child with severe neonatal thrombotic and hemorrhagic complications. Similar to fibrinogen Niigata, the reported mutation affects the C-terminal region of the Bβ chain coiled-coil. The authors speculate that extra oligosaccharides, introducing strong negative electric charges, may arise and affect lateral association of fibrin protofibrils and/or fiber packing (Asselta et al., 2015). Such phenomena were reported in other hyperglycosylated fibrinogens (Marchi et al., 2004; Woodhead et al., 1996). An additional N-glycosylation was also identified in the C-terminus of the γ-chain (γ380Lys to Asn), known as fibrinogen Kaiserslautern, proximal to the key residue involved in the formation of *a* polymerization pocket and the high-affinity calcium binding site. While the mutation does not disrupt these functional sites directly, the charge shift delays polymerization and reduces fiber diameter (Ridgway et al., 1997). Similarly, impaired polymerization of fibrin monomers was found in fibrinogen Asahi, in which a γ310Met to Thr substitution forms a consensus sequence for N-glycosylation at position 308Asn (Yamazumi et al., 1989).

The dysfibrinogenemia associated with liver disease has been further characterized by an increased sialic acid content, which directly correlated with prolonged thrombin time. Removal of the excess sialic acid restores normal thrombin time and fibrin monomer polymerization (Martinez et al., 1983; Martinez et al., 1978). Liver cirrhosis, a major risk factor for hemostatic imbalance, often leads to either bleeding or thrombosis (Driever and Lisman, 2023). The study of Gligorijević et al. analysed the fibrinogen glycosylation pattern by lectin-based protein microarray, together with the carbonylation pattern of the individual fibrinogen chains in cirrhotic patients. The results pointed to a typical increase in several carbohydrate moieties (Galβ-1,4GlcNAc, terminal α-2,3 Sia, and α1,3 Man), a decrease in core-fibrinogen fucosylation, and higher carbonylation susceptibility of the Aα chain (Gligorijević et al., 2018a). Sialic acid content of fibrin (ogen) in cirrhosis affects polymerization rates and results in decreased permeability, but the structure of a matured clot may also be affected by other PTMs than only sialylation of fibrinogen (Driever and Lisman, 2023; Gligorijević et al., 2018a; Hugenholtz et al., 2016). Liver cirrhosis (LC) is the main risk factor for hepatocellular carcinoma (HCC), the most common type of liver cancer. Nager et al. analysed samples from HCC patients, LC patients, and healthy donors to determine the glycosylation and phosphorylation patterns of fibrinogen and to relate those to pathological states. While glycosylation and phosphorylation patterns could distinguish patients from healthy individuals, they could not reliably differentiate between LC and HCC. Notably, Aα phosphorylation was reduced in HCC, while O-glycans were decreased in LC. Sialylation and fucosylation of N-glycans in the Bβ and γ chains increased in both disease groups (Nagel et al., 2018). Increased fibrinogen sialic acid content associated with thrombotic tendency and normal liver function was also found in a 25-year-old man case report, probably a new variant of congenital dysfibrinogenaemia (fibrinogen Copenhagen II) (Hansen and Schousboe, 1984), in pregnancy and in patients undergoing fibrate therapy (Maghzal et al., 2005).

As previously discussed, the analysis of protein glycosylation has received more attention due to the growing body of evidence that shows that PTMs might be useful biomarkers for CVDs and aid in uncovering the mechanisms involved in their development and progression (Hoshi et al., 2024). Among plasma proteins, there is an overwhelming interest in the characterization of fibrinogen profile glycosylation and its relevance as a biomarker in different diseases (Adamczyk et al., 2013). Both single-nucleotide polymorphisms (SNPs) and PTMs are associated with fibrinogen levels, clotting behaviour and cardiovascular risk (Nagel and Meyer, 2014).

A recent study assessed the diagnostic potential of fibrinogen N-glycan profiles in AF patients undergoing catheter ablation. Three low-abundance glycopeptides from the γ-chain were significantly reduced in AF patients compared to controls (Šoić et al., 2025). A rise in asialylated glycoforms may underlie the prothrombotic state observed in AF (Šoić et al., 2025; Dang et al., 1989).

Calcium-binding at low-affinity sialic acid sites on fibrinogen may facilitate polymerization, while high-affinity Ca^2+^ sites in the D-domain influence fibrin tertiary structure (Dang et al., 1989; Okude et al., 1993). Conversely, an abnormally high degree of sialylated fibrinogen in patients with COVID-19 (Moiseiwitsch et al., 2022) and chronic thromboembolic pulmonary hypertension (CTEPH) (Morris et al., 2007)seems to contribute to thrombotic clinical features in these diseases. Fibrin clots from COVID-19 patients were shown to be significantly stiffer and denser than clots made using fibrinogen from non-infected subjects, and these differences are at least in part mediated by differential sialylation (Moiseiwitsch et al., 2022). Morris et al. reported a fibrinogen variant in a CTEPH patient, which supports the hypothesis that an abnormally high degree of fibrinogen disialylation can influence clot susceptibility to plasmin-mediated lysis (Morris et al., 2007).

Glycosylated fibrinogen is especially relevant to the pathogenesis of DM. Main results from Daugaard et al. showed that total fibrinogen and absolute levels of fibrinogen α_E_, fibrinogen γ′, and sialylated fibrinogen were higher at baseline in patients with DM who later experienced strokes (Daugaard et al., 2025). Similar findings were observed in obese patients, while in bariatric surgery, sialylated fibrinogen levels were lower (Bødker Pedersen et al., 2025).

A high risk of cardiovascular complications has been observed in patients with end-stage renal disease (ESRD), and this is at least partly associated with delayed clot formation, increased clot strength, and delayed clot lysis (Nunns et al., 2017). Two recent studies from Baralić et al. aimed to examine fibrinogen glycosylation in patients with ESRD (Baralić et al., 2020; Baralić et al., 2023). Results from the first study imply that the fibrinogen A2BG2 glycan increases, while FA2 decreases. These changes were most prominent in the γ-chain, and fucosylation was strongly associated with peritoneal membrane damage in patients on peritoneal dialysis (Baralić et al., 2020). A follow-up study suggested that mannose-rich glycans on fibrinogen may be predictive of all-cause and cardiovascular mortality in ESRD patients (Baralić et al., 2023).

The influence of aging on fibrinogen carbohydrate content was also investigated by isolating fibrinogen from plasma samples of healthy subjects aged 21 to 83. Lectin microarray analysis on fibrinogen demonstrated increased glycosylation of fibrinogen due to aging, with a predominant increase in high-mannose or hybrid type N-glycans, as well as tri-/tetraantennary complex N-glycans with greater content of galactose and N-acetylglucosamine residues. Glycosylation changes of fibrinogen in healthy aging most likely affect clot structure, resulting in a more densely packed network, and function, namely clotting time (Gligorijević et al., 2018b).

Palomino et al. recently compared fetal and adult fibrinogen, identifying 39 glycans in fetal fibrinogen and increased glycosylation and sialylation, particularly in the Bβ chain, supporting earlier findings that knob ‘B’ interactions are more prominent in fetal fibrin formation (Palomino et al., 2024).

Fibrinogen O-linked oligosaccharides are less well described (Zauner et al., 2012). The first indications for mammalian fibrinogen O-glycosylation came from a report using fibrinogen in lectin binding studies (L'Hôte et al., 1996). Recent evidence for O-glycosylation of human fibrinogen originates from a comparative urine metabolomics analysis from patients suffering from urinary tract infection (UTI) versus controls. The increased release of fibrinogen fragments in urine is a response to an infection or infection-related kidney damage. Using a non-targeted exploratory UPLC–MS–based approach for the investigation of UTI-related changes in urine, authors have characterized a unique C-terminal O-glycopeptide of the human fibrinogen α-chain. An unusual O-glycosylation might be interpreted as an indication of the extrahepatic origin of the fragments. However, the clinical significance of this finding should be explored (Pacchiarotta et al., 2012).

### 4.2 Fibrinogen post translational modifications: glycation

As previously discussed, MGO is a main player in oxidative stress, inflammation, aging, and hyperglycemia. In hyperglycemic conditions, non-enzymatic glycation of fibrinogen plays a crucial role in the pathogenesis of micro- and macrovascular complications, especially in pathological conditions such as DM and atherosclerosis (Rehman et al., 2021a). Additionally, MGO induces structural alterations in fibrinogen through glycation, which can lead to an autoimmune response via the generation of neoepitopes on protein molecules (Rehman et al., 2021a).

*In vitro* studies have consistently shown that hyperglycemia leads to structural and functional alterations in fibrinogen (Robi et al., 1991; Brownlee et al., 1983; Khanam et al., 2021; Svensson et al., 2012; Norton et al., 2017; Mirmiranpour et al., 2012; Hood et al., 2018; Krantz et al., 1987) and the extent of glycation depends on the incubation time, temperature, and pH (McVerry et al., 1981; Mirshahi et al., 1987). To mimic hyperglycemic conditions, fibrinogen has been incubated with D-ribose (Khanam et al., 2021), glucose (Svensson et al., 2012; Norton et al., 2017; Mirmiranpour et al., 2012; Hood et al., 2018; Krantz et al., 1987), or MGO (Ahmad et al., 2024; Perween et al., 2022; Rehman et al., 2020; Perween et al., 2019; Alouffi et al., 2022a). To elucidate D-ribose-mediated glycation damage, fibrinogen was treated with progressively increasing concentrations of D-ribose and was analysed by UV-vis fluorescence, circular dichroism (CD), scanning electron microscopy (SEM), and Fourier transform infrared (FTIR) spectroscopy. Also, protein aggregation and fibril formation were confirmed by thioflavin T (ThT) and Congo red assay (Khanam et al., 2021).

Studies examining morphological changes in glucose-incubated fibrin matrices were conducted to gain insight into the mechanisms underlying the hypercoagulable state associated with hyperglycemia (Norton et al., 2017; Hood et al., 2018). One study demonstrated that glycated fibrin structures exhibit greater aggregation and anisotropy than their unglycated counterparts. However, extending the glucose incubation period, simulating physiological plasma glucose levels, resulted in fibrin clot structures that were less aggregated and more isotropic than those formed from non-incubated fibrinogen (Norton et al., 2017). By measurement of clot fractal dimension (d_f_), which associates higher d_f_ with a denser fibrin clot structure, Hood et al. reported that the 10.0 mmol/L glucose concentration condition produced the densest formed fibers, compared to 6.0 mmol/L and 0.0 mmol/L glucose concentration. Kinetic measurements of fibrinogen also suggest that increased glucose concentration slows clotting time (Hood et al., 2018). Additionally, fibrinogen glycation has been reported to significantly decrease clot susceptibility to plasmin-induced degradation (Brownlee et al., 1983; Mirshahi et al., 1987). Interestingly, Svensson et al. reported two glycated lysine (GlcK-133 in the β-chain and GlcK-75 in the γ-chain) within the ‘‘plasmin-sensitive’’ coiled-coil–coil regions. Of particular interest, GlcK-133 is located just two residues away from the known plasmin cleavage site at K-130, suggesting that glycation at this position could interfere with normal fibrinolysis (Svensson et al., 2012).

Under *in vitro* conditions, MGO is widely used to study hyperglycemia-induced non-enzymatic glycation of proteins (Rehman et al., 2020; Perween et al., 2019; Banerjee, 2021; Ahmed et al., 2018). Fibrinogen glycation is typically achieved by incubation of human fibrinogen with fixed (Perween et al., 2022; Rehman et al., 2020) or increased concentrations of MGO (Ahmad et al., 2024; Perween et al., 2019; Alouffi et al., 2022a) for varying incubation times (from 0 to 14 days) at 37 °C to mimic hyperglycemic conditions seen in DM.

MGO altered the tertiary and secondary structure of fibrinogen. While glycation primarily targets lysine and arginine residues, histidine and cysteine can also be affected (Perween et al., 2022). MGO-fibrinogen interaction was characterized using UV–Visible spectroscopy, revealing concentration- and time-dependent hyperchromicity in the absorbance profiles (Ahmad et al., 2024; Perween et al., 2019; Alouffi et al., 2022a). Alterations in the fluorescence spectra are widely used to examine the extent of changes in the aromatic amino acids tyrosine (Tyr), tryptophan (Trp), and phenylalanine (Phe). Results show a significant decrease in intrinsic fluorescence spectra of MGO-fibrinogen molecules in comparison to their native analogues (Ahmad et al., 2024; Rehman et al., 2020; Perween et al., 2019; Alouffi et al., 2022a). FTIR analysis revealed a notable shift in the amide I band from 1642 to 1646 cm^-1^ in MGO-modified fibrinogen, attributed to C=O stretching and altered hydrophilic interactions, potentially disrupting hydrophobic protein regions (Perween et al., 2022; Rehman et al., 2020; Perween et al., 2019; Alouffi et al., 2022a). Far-UV CD spectra showed a marked reduction in ellipticity, indicating a loss of native α-helical content and an increase in β-sheet structure, features associated with protein misfolding and aggregation (Ahmad et al., 2024; Perween et al., 2022; Rehman et al., 2020; Perween et al., 2019; Alouffi et al., 2022a). Taken together, fluorescence, FTIR, and CD results suggested that glycation affects the secondary and tertiary structure of fibrinogen. Amyloid-like aggregates were confirmed by ThT and Congo red assay (Rehman et al., 2020; Perween et al., 2019; Alouffi et al., 2022a), and the typical feature observed under SEM and TEM strongly supports the microstructure of the aggregates (Ahmad et al., 2024; Perween et al., 2022; Perween et al., 2019; Alouffi et al., 2022a).

DM is characterized by chronic hyperglycemia, which increases cardiovascular risk. Alterations in fibrin structure due to fibrinogen glycation may contribute to this risk (Dunn et al., 2005; Bembde, 2012). Diabetic subjects have been shown to exhibit altered fibrin network structures, with non-enzymatic glycation of fibrinogen, driven by elevated blood glucose, proposed as a key contributing mechanism (Mirmiranpour et al., 2012; Dunn et al., 2005; Bembde, 2012; Dunn et al., 2006; Pieters et al., 2008; Li et al., 2016; Luzak et al., 2020; Pieters et al., 2006; Jörneskog et al., 2003). Several studies have investigated the effect of fibrinogen glycation on fibrin structure through *in vitro* analysis of diabetic patients’ purified fibrinogen (Dunn et al., 2005; Dunn et al., 2006; Pieters et al., 2008; Li et al., 2016; Luzak et al., 2020; Jörneskog et al., 2003).

Importantly, diabetic patients undergoing glycemic control treatment show a reduction in fibrinogen glycation (Pieters et al., 2008; Li et al., 2016; Pieters et al., 2006; Pieters et al., 2007). Moreover, higher plasma fibrinogen levels were found in T2DM patients as compared to controls (Bembde, 2012; Pieters et al., 2006), and hyperfibrinogenaemia seems to be correlated with HbA1c values (Bembde, 2012; Pieters et al., 2007). Notably, glycated fibrinogen has shown potential as a complementary marker to HbA1c for monitoring glycemic control (Pieters et al., 2007).

In diabetic subjects, the fibrin network and the fibrin gel porosity are impaired, potentially due to fibrinogen glycation, which alters both structural and functional properties. Li et al. used a combined atomic force microscopy/fluorescence microscopy technique to determine the mechanical properties of individual fibrin fibers formed from diabetic plasma. Their findings showed no direct correlation between fibrinogen glycation and fibrin fiber extensibility, modulus, and stress relaxation, whereas the fiber modulus, Y, strongly decreases with increasing fiber diameter, D. The strong dependence of the Y on D is very unusual and has interesting and significant consequences for whole-clot properties, and especially for the internal structure and lateral assembly of fibrin fibers (Li et al., 2016).

Clot structure can also be assessed by turbidity, permeability, confocal microscopy, and SEM. One *in vitro* study using purified fibrinogen found that clots from diabetic subjects were denser and less porous than those from control subjects (Dunn et al., 2005). Conversely, another study showed that the fiber diameter of the clots from diabetic and non-diabetic subjects was similar both at baseline and after achieving glycaemic control, though a slight increase in the proportion of thicker fibers was noted in diabetic clots post-treatment (Pieters et al., 2008). Liquid permeation studies further revealed that the fibrin gel permeability coefficient (Ks) is significantly reduced in diabetic patients, indicating a tighter and less permeable fibrin network (Dunn et al., 2005; Pieters et al., 2008; Jörneskog et al., 2003).

Turbidity measurements were used to assess polymerization kinetics, including the lag phase, maximum slope (Vmax), and maximum absorbance (MaxAbs). However, the results across studies were inconclusive. Regarding the lag time (the time required for fibrin fibers to grow sufficiently to allow absorbance detection), one study reported no difference between diabetic and non-diabetic subjects (Pieters et al., 2008), while another reported a significantly shorter lag time in clots from diabetic patients (Dunn et al., 2005). Conflicting results were also observed for Vmax, a measure of the rate of lateral aggregation. Pieters et al. reported that the Vmax in diabetic subjects was significantly higher, and it decreased after glycaemic control (Pieters et al., 2008). Conversely, Luzak et al. observed that Vmax in the T2DM plasma clots was significantly lower than in non-diabetic controls. The same tendency was observed in the glucose-treated fibrinogen compared to the control protein (Luzak et al., 2020). No difference in the MaxAbs (average cross-sectional area of fibers) between diabetic subjects and controls was reported in two studies (Pieters et al., 2008; Luzak et al., 2020). According to Dunn et al., diabetic clots achieved a greater MaxAbs at full polymerisation (Dunn et al., 2005). Some authors speculate that it seems as though porosity, compaction, and kinetics of clot formation are more related to fibrinogen concentration than fibrinogen glycation in this model (Pieters et al., 2006).

Altered fibrin network formation may contribute to decreased fibrinolysis. Diabetic fibrin clots have been shown to exhibit significantly slower lysis rates compared to those from non-diabetic individuals, accompanied by reduced plasmin generation (Dunn et al., 2006). Notably, the achievement of glycaemic control and decreased fibrinogen glycation levels improves lysis rates in a purified fibrinogen model (Pieters et al., 2008). Interestingly, Mirmiranpour et al. investigated the effects of Lys supplementation in conventional T2DM treatment. Their results demonstrated that Lys, as an inhibitor of glycation, can be effective in the reduction of fibrinogen’s non-enzymatic glycation and the rectification of its structure and function. *In vivo*, patients receiving standard therapy with metformin and glibenclamide showed reduced fibrinogen activity; however, this decrease was significantly attenuated in the group receiving additional Lys supplementation (Mirmiranpour et al., 2012).

Structural modifications induced by glycation may also lead to the formation of neoepitopes capable of triggering immune responses. Glycated proteins elicit powerful and specific immunological responses, resulting in the production of antibodies (Rehman et al., 2021a; Siddiqui et al., 2019). Alouffi et al. show that immunization of rabbits with fibrinogen molecule glycated with D-ribose (Rb-gly-Fb) significantly upregulated the expression of TNF-α, IL-6, IL-1β, and IFN-γ mRNAs, indicative of the inflammatory response (Alouffi et al., 2022b). Consequently, autoantibodies against glycated fibrinogen have been proposed as a potential biomarker in early diagnosis of diabetes mellitus, but also in its associated secondary disorders (Rehman et al., 2021b).

## 5 Discussion and concluding remarks

CVDs remain the leading cause of morbidity and mortality worldwide. Beyond traditional risk factors, increasing attention is being directed toward PTMs of fibrinogen, particularly glycosylation and glycation, which profoundly influence fibrin clot properties. Evidence indicates that carbohydrate modifications alter fibrin polymerization, fiber thickness, clot density, and susceptibility to lysis, with glycosylation generally delaying polymerization and reducing fiber diameter, while glycation promotes aggregation, α-helix to β-sheet transitions, and impaired fibrinolysis.

Altered glycosylation patterns have been reported in liver disease, atrial fibrillation, COVID-19, chronic thromboembolic pulmonary hypertension, diabetes, and end-stage renal disease, suggesting their potential as disease-specific biomarkers. Advances such as high-throughput LC-MS have enabled site-specific profiling of fibrinogen N-glycosylation, revealing associations with clinical and biochemical parameters and supporting its role in cardiovascular risk stratification.

Experimental models mimicking hyperglycemia show that glycated fibrinogen produces denser, less lysis-prone clots and may trigger immune responses leading to autoantibody formation, raising the possibility that it contributes to diabetes-associated complications. However, major methodological limitations persist: studies differ in the source of fibrinogen and in the type and concentration of glycating agents (MGO or glucose), use heterogeneous incubation times, and lack assay standardization. Defining experimental conditions that truly mimic the diabetic state remains a crucial challenge. In addition, functional data on glycated fibrinogen are still scarce, highlighting the need for more rigorous and systematic investigations.

Overall, fibrinogen glycosylation and glycation emerge as promising biomarkers and mechanistic contributors to thrombotic events. Further studies are required to clarify the clinical significance of glycated fibrinogen, especially in diabetes, and to translate these insights into diagnostic and therapeutic strategies for high-risk populations. Despite the promising potential of glycosylated and glycated fibrinogen as biomarkers, several barriers still limit their clinical application. These include the lack of assay standardization, limited sensitivity and specificity of available methods, and the high cost of advanced analytical platforms. Future strategies to overcome these obstacles may involve the development of high-throughput LC-MS workflows, more robust and cost-effective immunoassays, and validation through large multicenter cohort studies. Addressing these issues will be essential to translate fibrinogen PTM profiling into routine cardiovascular risk assessment.

## References

[B1] Adamczyk B. Struwe W. B. Ercan A. Nigrovic P. A. Rudd P. M. (2013). Characterization of fibrinogen glycosylation and its importance for serum/plasma N-glycome analysis. J. Proteome Res. 12, 444–454. 10.1021/pr300813h 23151259

[B2] Ahmad R. Warsi M. S. Abidi M. Habib S. Siddiqui S. Khan H. (2024). Structural perturbations induced by cumulative action of methylglyoxal and peroxynitrite on human fibrinogen: an *in vitro* and *in silico* approach. Spectrochim. Acta A Mol. Biomol. Spectrosc. 307, 123500. 10.1016/j.saa.2023.123500 37989033

[B3] Ahmed A. Shamsi A. Khan M. S. Husain F. M. Bano B. (2018). Methylglyoxal induced glycation and aggregation of human serum albumin: biochemical and biophysical approach. Int. J. Biol. Macromol. 113, 269–276. 10.1016/j.ijbiomac.2018.02.137 29481950

[B4] Akinkuolie A. O. Buring J. E. Ridker P. M. Mora S. (2014). A novel protein glycan biomarker and future cardiovascular disease events. J. Am. Heart Assoc. 3, e001221. 10.1161/JAHA.114.001221 25249300 PMC4323825

[B5] Alouffi S. Shahab U. Khan S. Khan M. Khanam A. Akasha R. (2022a). Glyoxal induced glycative insult suffered by immunoglobulin G and fibrinogen proteins: a comparative physicochemical characterization to reveal structural perturbations. Int. J. Biol. Macromol. 205, 283–296. 10.1016/j.ijbiomac.2022.02.093 35192903

[B6] Alouffi S. Khanam A. Husain A. Akasha R. Rabbani G. Ahmad S. (2022b). d-ribose-mediated glycation of fibrinogen: role in the induction of adaptive immune response. Chem. Biol. Interact. 367, 110147. 10.1016/j.cbi.2022.110147 36108717

[B7] Asselta R. Robusto M. Platé M. Santoro C. Peyvandi F. Duga S. (2015). Molecular characterization of 7 patients affected by dys- or hypo-dysfibrinogenemia: identification of a novel mutation in the fibrinogen Bbeta chain causing a gain of glycosylation. Thromb. Res. 136, 168–174. 10.1016/j.thromres.2015.05.007 26006300

[B8] Asselta R. Paraboschi E. M. Duga S. (2020). Hereditary hypofibrinogenemia with hepatic storage. Int. J. Mol. Sci. 21, 7830. 10.3390/ijms21217830 33105716 PMC7659954

[B9] Ballout R. A. Remaley A. T. (2020). GlycA: a new biomarker for systemic inflammation and cardiovascular disease (CVD) risk assessment. J. Lab. Precis. Med. 5, 17. 10.21037/jlpm.2020.03.03 32363327 PMC7194207

[B10] Banerjee S. (2021). Biophysical and mass spectrometry based characterization of methylglyoxal-modified myoglobin: role of advanced glycation end products in inducing protein structural alterations. Int. J. Biol. Macromol. 193, 2165–2172. 10.1016/j.ijbiomac.2021.11.047 34774865

[B11] Baralić M. Gligorijević N. Brković V. Katrlík J. Pažitná L. Šunderić M. (2020). Fibrinogen fucosylation as a prognostic marker of end-stage renal disease in patients on peritoneal dialysis. Biomolecules 10, 1165. 10.3390/biom10081165 32784866 PMC7466146

[B12] Baralić M. Pažitná L. Brković V. Laušević M. Gligorijević N. Katrlík J. (2023). Prediction of mortality in patients on peritoneal dialysis based on the fibrinogen mannosylation. Cells 12, 351. 10.3390/cells12030351 36766693 PMC9913213

[B13] Becatti M. Marcucci R. Bruschi G. Taddei N. Bani D. Gori A. M. (2014). Oxidative modification of fibrinogen is associated with altered function and structure in the subacute phase of myocardial infarction. Arterioscler. Thromb. Vasc. Biol. 34, 1355–1361. 10.1161/ATVBAHA.114.303785 24790138

[B14] Becatti M. Emmi G. Silvestri E. Bruschi G. Ciucciarelli L. Squatrito D. (2016). Neutrophil activation promotes fibrinogen oxidation and thrombus formation in behçet disease. Circulation 133, 302–311. 10.1161/CIRCULATIONAHA.115.017738 26585672

[B15] Becatti M. Mannucci A. Argento F. R. Gitto S. Vizzutti F. Marra F. (2020). Super-resolution microscopy reveals an altered fibrin network in cirrhosis: the key role of oxidative stress in fibrinogen structural modifications. Antioxidants (Basel) 9, 737. 10.3390/antiox9080737 32806658 PMC7464401

[B16] Becatti M. Emmi G. Bettiol A. Mannucci A. Argento F. R. Fini E. (2025). ROS-induced modifications of fibrin clots connect immune responses to atherothrombosis in systemic lupus erythematosus. Arthritis Rheumatol., art.43371. 10.1002/art.43371 40897511 PMC12936896

[B17] Bembde A. S. (2012). A study of plasma fibrinogen level in type-2 diabetes mellitus and its relation to glycemic control. Indian J. Hematol. Blood Transfus. 28, 105–108. 10.1007/s12288-011-0116-9 23730017 PMC3332265

[B18] Bettiol A. Argento F. R. Fini E. Bello F. Di Scala G. Taddei N. (2023). ROS-driven structural and functional fibrinogen modifications are reverted by interleukin-6 inhibition in Giant Cell Arteritis. Thromb. Res. 230, 1–10. 10.1016/j.thromres.2023.08.011 37598635

[B19] Birukov A. Cuadrat R. Polemiti E. Eichelmann F. Schulze M. B. (2021). Advanced glycation end-products, measured as skin autofluorescence, associate with vascular stiffness in diabetic, pre-diabetic and normoglycemic individuals: a cross-sectional study. Cardiovasc Diabetol. 20, 110. 10.1186/s12933-021-01296-5 34176469 PMC8236143

[B20] Bødker Pedersen N. Münster A. B. Munk Lauridsen M. Palarasah Y. Wernberg C. W. Ladegaard Grønkjær L. (2025). Association of fibrinogen variants with severity of obesity and metabolic liver disease: 2-year follow-up after bariatric surgery. Thromb. Haemost. 10.1055/a-2615-4682 40389229

[B21] Brownlee M. Vlassara H. Cerami A. (1983). Nonenzymatic glycosylation reduces the susceptibility of fibrin to degradation by plasmin. Diabetes 32, 680–684. 10.2337/diab.32.7.680 6222931

[B22] Casini A. Undas A. Palla R. Thachil J. de Moerloose P. Fibrinogen S. o.F. X. a. (2018). Diagnosis and classification of congenital fibrinogen disorders: communication from the SSC of the ISTH. J. Thromb. Haemost. 16, 1887–1890. 10.1111/jth.14216 30076675

[B23] Cellai A. P. Lami D. Antonucci E. Fiorillo C. Becatti M. Olimpieri B. (2013). Fibrinolytic inhibitors and fibrin characteristics determine a hypofibrinolytic state in patients with pulmonary embolism. Thromb. Haemost. 109, 565–567. 10.1160/TH12-09-0648 23306795

[B24] Chatham J. C. Patel R. P. (2024). Protein glycosylation in cardiovascular health and disease. Nat. Rev. Cardiol. 21, 525–544. 10.1038/s41569-024-00998-z 38499867

[B25] Clerc F. Reiding K. R. Jansen B. C. Kammeijer G. S. Bondt A. Wuhrer M. (2016). Human plasma protein N-glycosylation. Glycoconj J. 33, 309–343. 10.1007/s10719-015-9626-2 26555091 PMC4891372

[B26] Connelly M. A. Gruppen E. G. Otvos J. D. Dullaart R. P. F. (2016). Inflammatory glycoproteins in cardiometabolic disorders, autoimmune diseases and cancer. Clin. Chim. Acta 459, 177–186. 10.1016/j.cca.2016.06.012 27312321

[B27] Czuba L. C. Hillgren K. M. Swaan P. W. (2018). Post-translational modifications of transporters. Pharmacol. Ther. 192, 88–99. 10.1016/j.pharmthera.2018.06.013 29966598 PMC6263853

[B28] Damiana T. Damgaard D. Sidelmann J. J. Nielsen C. H. de Maat M. P. M. Münster A. B. (2020). Citrullination of fibrinogen by peptidylarginine deiminase 2 impairs fibrin clot structure. Clin. Chim. Acta 501, 6–11. 10.1016/j.cca.2019.10.033 31730822

[B29] Dang C. V. Shin C. K. Bell W. R. Nagaswami C. Weisel J. W. (1989). Fibrinogen sialic acid residues are low affinity calcium-binding sites that influence fibrin assembly. J. Biol. Chem. 264, 15104–15108. 10.1016/s0021-9258(18)63817-7 2549051

[B30] Dashti H. Pabon Porras M. A. Mora S. (2021). Glycosylation and cardiovascular diseases. Adv. Exp. Med. Biol. 1325, 307–319. 10.1007/978-3-030-70115-4_15 34495542

[B31] Daugaard N. Bladbjerg E. M. Lundsgaard Svane H. M. Thomsen R. W. Nielsen J. S. Palarasah Y. (2025). Association of fibrinogen α_E_, fibrinogen γ', and sialylated fibrinogen with development of ischemic stroke in patients with recently diagnosed type 2 diabetes. J. Thromb. Haemost. 23, 2213–2225. 10.1016/j.jtha.2025.03.023 40187413

[B32] de Moerloose P. Casini A. Neerman-Arbez M. (2013). Congenital fibrinogen disorders: an update. Semin. Thromb. Hemost. 39, 585–595. 10.1055/s-0033-1349222 23852822

[B33] Demina E. P. Smutova V. Pan X. Fougerat A. Guo T. Zou C. (2021). Neuraminidases 1 and 3 trigger atherosclerosis by desialylating low-density lipoproteins and increasing their uptake by macrophages. J. Am. Heart Assoc. 10, e018756. 10.1161/JAHA.120.018756 33554615 PMC7955353

[B34] Di Angelantonio E. Gao P. Khan H. Butterworth A. S. Wormser D. Kaptoge S. (2014). Glycated hemoglobin measurement and prediction of cardiovascular disease. JAMA 311, 1225–1233. 10.1001/jama.2014.1873 24668104 PMC4386007

[B35] Dinu M. Whittaker A. Pagliai G. Giangrandi I. Colombini B. Gori A. M. (2018). A khorasan wheat-based replacement diet improves risk profile of patients with nonalcoholic fatty liver disease (nafld): a randomized clinical trial. J. Am. Coll. Nutr. 37, 508–514. 10.1080/07315724.2018.1445047 29652567

[B36] Dobson D. A. Fish R. J. de Vries P. S. Morrison A. C. Neerman-Arbez M. Wolberg A. S. (2024). Regulation of fibrinogen synthesis. Thromb. Res. 242, 109134. 10.1016/j.thromres.2024.109134 39216273 PMC11381137

[B37] Dozio E. Massaccesi L. Corsi Romanelli M. M. (2021). Glycation and glycosylation in cardiovascular remodeling: focus on advanced glycation end products and O-linked glycosylations as glucose-related pathogenetic factors and disease markers. J. Clin. Med. 10, 4792. 10.3390/jcm10204792 34682915 PMC8539574

[B38] Driever E. G. Lisman T. (2023). Fibrin clot properties and thrombus composition in cirrhosis. Res. Pract. Thromb. Haemost. 7, 100055. 10.1016/j.rpth.2023.100055 36798901 PMC9925609

[B39] Dunn E. J. Ariëns R. A. Grant P. J. (2005). The influence of type 2 diabetes on fibrin structure and function. Diabetologia 48, 1198–1206. 10.1007/s00125-005-1742-2 15864538

[B40] Dunn E. J. Philippou H. Ariëns R. A. Grant P. J. (2006). Molecular mechanisms involved in the resistance of fibrin to clot lysis by plasmin in subjects with type 2 diabetes mellitus. Diabetologia 49, 1071–1080. 10.1007/s00125-006-0197-4 16538489

[B41] Duprez D. A. Otvos J. Sanchez O. A. Mackey R. H. Tracy R. Jacobs D. R. (2016). Comparison of the predictive value of GlycA and other biomarkers of inflammation for total death, incident cardiovascular events, noncardiovascular and noncancer inflammatory-related events, and total cancer events. Clin. Chem. 62, 1020–1031. 10.1373/clinchem.2016.255828 27173011

[B42] Duval C. Ariëns R. A. S. (2017). Fibrinogen splice variation and cross-linking: effects on fibrin structure/function and role of fibrinogen γ' as thrombomobulin II. Matrix Biol. 60-61, 8–15. 10.1016/j.matbio.2016.09.010 27784620

[B43] Emmi G. Bettiol A. Niccolai E. Ramazzotti M. Amedei A. Pagliai G. (2021). Butyrate-rich diets improve redox status and fibrin lysis in behçet's syndrome. Circ. Res. 128, 278–280. 10.1161/CIRCRESAHA.120.317789 33198585

[B44] Fini E. Argento F. R. Borghi S. Giurranna E. Nencini F. Cirillo M. (2024). Fibrinogen structural changes and their potential role in endometriosis-related thrombosis. Antioxidants (Basel) 13, 1456. 10.3390/antiox13121456 39765785 PMC11673276

[B45] Fish R. J. Neerman-Arbez M. (2012). Fibrinogen gene regulation. Thromb. Haemost. 108, 419–426. 10.1160/TH12-04-0273 22836683

[B46] Gitto S. Fiorillo C. Argento F. R. Fini E. Borghi S. Falcini M. (2024). Oxidative stress-induced fibrinogen modifications in liver transplant recipients: unraveling a novel potential mechanism for cardiovascular risk. Res. Pract. Thromb. Haemost. 8, 102555. 10.1016/j.rpth.2024.102555 39309232 PMC11416524

[B47] Giurranna E. Nencini F. Borghi S. Barbaro I. Taddei N. Fiorillo C. (2025). Homocysteinylation of fibrinogen: a post-translational link to thrombosis. Int. J. Mol. Sci. 26, 5471. 10.3390/ijms26125471 40564934 PMC12193665

[B48] Gligorijević N. Minić S. Križáková M. Katrlík J. Nedić O. (2018a). Structural changes of fibrinogen as a consequence of cirrhosis. Thromb. Res. 166, 43–49. 10.1016/j.thromres.2018.04.005 29655002

[B49] Gligorijević N. Zámorová Križáková M. Penezić A. Katrlík J. Nedić O. (2018b). Structural and functional changes of fibrinogen due to aging. Int. J. Biol. Macromol. 108, 1028–1034. 10.1016/j.ijbiomac.2017.11.016 29137999

[B50] Güven B. Can M. (2024). Fibrinogen: structure, abnormalities and laboratory assays. Adv. Clin. Chem. 120, 117–143. 10.1016/bs.acc.2024.03.004 38762239

[B51] Hansen M. S. Schousboe I. (1984). An abnormal fibrinogen (Copenhagen II) with increased sialic acid content associated with thrombotic tendency and normal liver function. Scand. J. Haematol. 33, 9–14. 10.1111/j.1600-0609.1984.tb02203.x 6205441

[B52] Hellwig M. Diel P. Eisenbrand G. Grune T. Guth S. Henle T. (2024). Dietary glycation compounds - implications for human health. Crit. Rev. Toxicol. 54, 485–617. 10.1080/10408444.2024.2362985 39150724

[B53] Hevér H. Darula Z. Medzihradszky K. F. (2019). Characterization of site-specific N-glycosylation. Methods Mol. Biol. 1934, 93–125. 10.1007/978-1-4939-9055-9_8 31256376

[B54] Hood J. E. Yesudasan S. Averett R. D. (2018). Glucose concentration affects fibrin clot structure and morphology as evidenced by fluorescence imaging and molecular simulations. Clin. Appl. Thromb. Hemost. 24, 104S–116S. 10.1177/1076029618792304 30114949 PMC6714860

[B55] Hoshi R. A. Plavša B. Liu Y. Trbojević-Akmačić I. Glynn R. J. Ridker P. M. (2024). N-glycosylation profiles of immunoglobulin G and future cardiovascular events. Circ. Res. 134, e3–e14. 10.1161/CIRCRESAHA.123.323623 38348651 PMC10923145

[B56] Huang Y. Zhang H. L. Li Z. L. Du T. Chen Y. H. Wang Y. (2021). FUT8-mediated aberrant N-glycosylation of B7H3 suppresses the immune response in triple-negative breast cancer. Nat. Commun. 12, 2672. 10.1038/s41467-021-22618-x 33976130 PMC8113546

[B57] Hugenholtz G. C. Macrae F. Adelmeijer J. Dulfer S. Porte R. J. Lisman T. (2016). Procoagulant changes in fibrin clot structure in patients with cirrhosis are associated with oxidative modifications of fibrinogen. J. Thromb. Haemost. 14, 1054–1066. 10.1111/jth.13278 26833718

[B58] Jamieson J. C. Kaplan H. A. Woloski B. M. Hellman M. Ham K. (1983). Glycoprotein biosynthesis during the acute-phase response to inflammation. Can. J. Biochem. Cell Biol. 61, 1041–1048. 10.1139/o83-133 6627106

[B59] Jayaprakash N. G. Surolia A. (2017). Role of glycosylation in nucleating protein folding and stability. Biochem. J. 474, 2333–2347. 10.1042/BCJ20170111 28673927

[B60] Jörneskog G. Hansson L. O. Wallen N. H. Yngen M. Blombäck M. (2003). Increased plasma fibrin gel porosity in patients with Type I diabetes during continuous subcutaneous insulin infusion. J. Thromb. Haemost. 1, 1195–1201. 10.1046/j.1538-7836.2003.00301.x 12871319

[B61] Kattula S. Byrnes J. R. Wolberg A. S. (2017). Fibrinogen and fibrin in hemostasis and thrombosis. Arterioscler. Thromb. Vasc. Biol. 37, e13–e21. 10.1161/ATVBAHA.117.308564 28228446 PMC5324399

[B62] Khanam A. Alouffi S. Rehman S. Ansari I. A. Shahab U. Ahmad S. (2021). An *in vitro* approach to unveil the structural alterations in d-ribose induced glycated fibrinogen. J. Biomol. Struct. Dyn. 39, 5209–5223. 10.1080/07391102.2020.1802339 32772827

[B63] Kobata A. (1992). Structures and functions of the sugar chains of glycoproteins. Eur. J. Biochem. 209, 483–501. 10.1111/j.1432-1033.1992.tb17313.x 1358608

[B64] Krantz S. Lober M. Thiele M. Teuscher E. (1987). Properties of *in vitro* nonenzymatically glycated plasma fibrinogens. Exp. Clin. Endocrinol. 90, 37–45. 10.1055/s-0029-1210670 3666059

[B65] Kurien B. T. Hensley K. Bachmann M. Scofield R. H. (2006). Oxidatively modified autoantigens in autoimmune diseases. Free Radic. Biol. Med. 41, 549–556. 10.1016/j.freeradbiomed.2006.05.020 16863987

[B66] L'Hôte C. Berger S. Karamanos Y. (1996). O-glycosylation of fibrinogen from different mammalian species as revealed by the binding of *Escherichia coli* biotinylated lectins. Thromb. Haemost. 76, 710–714. 10.1055/s-0038-1650648 8950778

[B67] Langer B. G. Weisel J. W. Dinauer P. A. Nagaswami C. Bell W. R. (1988). Deglycosylation of fibrinogen accelerates polymerization and increases lateral aggregation of fibrin fibers. J. Biol. Chem. 263, 15056–15063. 10.1016/s0021-9258(18)68145-1 3170575

[B68] Lawler P. R. Akinkuolie A. O. Chandler P. D. Moorthy M. V. Vandenburgh M. J. Schaumberg D. A. (2016). Circulating N-linked glycoprotein acetyls and longitudinal mortality risk. Circ. Res. 118, 1106–1115. 10.1161/CIRCRESAHA.115.308078 26951635 PMC4836171

[B69] Li W. Sigley J. Pieters M. Helms C. C. Nagaswami C. Weisel J. W. (2016). Fibrin fiber stiffness is strongly affected by fiber diameter, but not by fibrinogen glycation. Biophys. J. 110, 1400–1410. 10.1016/j.bpj.2016.02.021 27028649 PMC4816776

[B70] Lin Z. He J. Yuan S. Song C. Bian X. Yang M. (2024). Hemoglobin glycation index and cardiovascular outcomes in patients with diabetes and coronary artery disease: insights from a large cohort study. Nutr. Diabetes 14, 69. 10.1038/s41387-024-00318-x 39191777 PMC11349977

[B71] Lipinski B. Pretorius E. (2012). Hydroxyl radical-modified fibrinogen as a marker of thrombosis: the role of iron. Hematology 17, 241–247. 10.1179/1607845412Y.0000000004 22889519

[B72] Lu X. Wang L. Wang M. Li Y. Zhao Q. Shi Y. (2023). Association between immunoglobulin G N-glycosylation and lupus nephritis in female patients with systemic lupus erythematosus: a case-control study. Front. Immunol. 14, 1257906. 10.3389/fimmu.2023.1257906 37809087 PMC10552529

[B73] Luévano-Contreras C. Gómez-Ojeda A. Macías-Cervantes M. H. Garay-Sevilla M. E. (2017). Dietary advanced glycation end products and cardiometabolic risk. Curr. Diab Rep. 17, 63. 10.1007/s11892-017-0891-2 28695383

[B74] Luzak B. Boncler M. Kosmalski M. Mnich E. Stanczyk L. Przygodzki T. (2020). Fibrinogen glycation and presence of glucose impair fibrin polymerization-an *in vitro* study of isolated fibrinogen and plasma from patients with diabetes mellitus. Biomolecules 10, 877. 10.3390/biom10060877 32517350 PMC7356284

[B75] Ma B. Chen H. Gong J. Liu W. Wei X. Zhang Y. (2024). Enhancing protein solubility via glycosylation: from chemical synthesis to machine learning predictions. Biomacromolecules 25, 3001–3010. 10.1021/acs.biomac.4c00134 38598264

[B76] Maghzal G. J. Brennan S. O. George P. M. (2005). The sialic acid content of fibrinogen decreases during pregnancy and increases in response to fibrate therapy. Thromb. Res. 115, 293–299. 10.1016/j.thromres.2004.08.013 15668189

[B77] Marchi R. Arocha-Piñango C. L. Nagy H. Matsuda M. Weisel J. W. (2004). The effects of additional carbohydrate in the coiled-coil region of fibrinogen on polymerization and clot structure and properties: characterization of the homozygous and heterozygous forms of fibrinogen Lima (Aalpha Arg141Ser with extra glycosylation). J. Thromb. Haemost. 2, 940–948. 10.1111/j.1538-7836.2004.00730.x 15140130

[B78] Martin S. S. Aday A. W. Allen N. B. Almarzooq Z. I. Anderson C. A. M. Arora P. (2025). 2025 heart disease and stroke statistics: a report of us and global data from the American heart association. Circulation 151, e41–e660. 10.1161/CIR.0000000000001303 39866113 PMC12256702

[B79] Martinez J. Palascak J. E. Kwasniak D. (1978). Abnormal sialic acid content of the dysfibrinogenemia associated with liver disease. J. Clin. Invest 61, 535–538. 10.1172/JCI108964 621288 PMC372564

[B80] Martinez J. Keane P. M. Gilman P. B. Palascak J. E. (1983). The abnormal carbohydrate composition of the dysfibrinogenemia associated with liver disease. Ann. N. Y. Acad. Sci. 408, 388–396. 10.1111/j.1749-6632.1983.tb23259.x 6575696

[B81] McVerry B. A. Thorpe S. Joe F. Gaffney P. Huehns E. R. (1981). Non-enzymatic glucosylation of fibrinogen. Haemostasis 10, 261–270. 10.1159/000214409 7274778

[B82] Menni C. Gudelj I. Macdonald-Dunlop E. Mangino M. Zierer J. Bešić E. (2018). Glycosylation profile of immunoglobulin G is cross-sectionally associated with cardiovascular disease risk score and Subclinical atherosclerosis in two independent cohorts. Circ. Res. 122, 1555–1564. 10.1161/CIRCRESAHA.117.312174 29535164 PMC5970566

[B83] Mirmiranpour H. Bathaie S. Z. Khaghani S. Nakhjavani M. Kebriaeezadeh A. (2012). Investigation of the mechanism(s) involved in decreasing increased fibrinogen activity in hyperglycemic conditions using L-lysine supplementation. Thromb. Res. 130, e13–e19. 10.1016/j.thromres.2012.04.010 22575419

[B84] Mirshahi M. Soria J. Soria C. Bertrand O. Basdevant A. (1987). Glycosylation of human fibrinogen and fibrin *in vitro*. Its consequences on the properties of fibrin(ogen). Thromb. Res. 48, 279–289. 10.1016/0049-3848(87)90440-3 3124289

[B85] Moiseiwitsch N. Zwennes N. Szlam F. Sniecinski R. Brown A. (2022). COVID-19 patient fibrinogen produces dense clots with altered polymerization kinetics, partially explained by increased sialic acid. J. Thromb. Haemost. 20, 2909–2920. 10.1111/jth.15882 36111490 PMC9537908

[B86] Morris T. A. Marsh J. J. Chiles P. G. Kim N. H. Noskovack K. J. Magana M. M. (2007). Abnormally sialylated fibrinogen gamma-chains in a patient with chronic thromboembolic pulmonary hypertension. Thromb. Res. 119, 257–259. 10.1016/j.thromres.2006.02.010 16626789

[B87] Nagel T. Meyer B. (2014). Simultaneous characterization of sequence polymorphisms, glycosylation and phosphorylation of fibrinogen in a direct analysis by LC-MS. Biochim. Biophys. Acta 1844, 2284–2289. 10.1016/j.bbapap.2014.09.021 25280394

[B88] Nagel T. Klaus F. Ibanez I. G. Wege H. Lohse A. Meyer B. (2018). Fast and facile analysis of glycosylation and phosphorylation of fibrinogen from human plasma-correlation with liver cancer and liver cirrhosis. Anal. Bioanal. Chem. 410, 7965–7977. 10.1007/s00216-018-1418-7 30397756

[B89] Neerman-Arbez M. (2006). Molecular basis of fibrinogen deficiency. Pathophysiol. Haemost. Thromb. 35, 187–198. 10.1159/000093566 16855369

[B90] Neerman-Arbez M. de Moerloose P. Casini A. (2016). Laboratory and genetic investigation of mutations accounting for congenital fibrinogen disorders. Semin. Thromb. Hemost. 42, 356–365. 10.1055/s-0036-1571340 27019463

[B91] Nencini F. Bettiol A. Argento F. R. Borghi S. Giurranna E. Emmi G. (2024). Post-translational modifications of fibrinogen: implications for clotting, fibrin structure and degradation. Mol. Biomed. 5, 45. 10.1186/s43556-024-00214-x 39477884 PMC11525374

[B92] Nencini F. Giurranna E. Borghi S. Taddei N. Fiorillo C. Becatti M. (2025a). Fibrinogen oxidation and thrombosis: Shaping structure and function. Antioxidants (Basel) 14, 390. 10.3390/antiox14040390 40298646 PMC12024030

[B93] Nencini F. Borghi S. Giurranna E. Barbaro I. Taddei N. Fiorillo C. (2025b). Reactive nitrogen species and fibrinogen: Exploring the effects of nitration on blood clots. Antioxidants (Basel) 14, 825. 10.3390/antiox14070825 40722929 PMC12291875

[B94] Norton D. G. Fan N. K. Goudie M. J. Handa H. Platt M. O. Averett R. D. (2017). Computational imaging analysis of glycated fibrin gels reveals aggregated and anisotropic structures. J. Biomed. Mater Res. A 105, 2191–2198. 10.1002/jbm.a.36074 28371216

[B95] Nunns G. R. Moore E. E. Chapman M. P. Moore H. B. Stettler G. R. Peltz E. (2017). The hypercoagulability paradox of chronic kidney disease: the role of fibrinogen. Am. J. Surg. 214, 1215–1218. 10.1016/j.amjsurg.2017.08.039 28951066 PMC5693753

[B96] Okude M. Yamanaka A. Morimoto Y. Akihama S. (1993). Sialic acid in fibrinogen: effects of sialic acid on fibrinogen-fibrin conversion by thrombin and properties of asialofibrin clot. Biol. Pharm. Bull. 16, 448–452. 10.1248/bpb.16.448 8364489

[B97] Otvos J. D. Shalaurova I. Wolak-Dinsmore J. Connelly M. A. Mackey R. H. Stein J. H. (2015). GlycA: a composite nuclear magnetic Resonance biomarker of systemic inflammation. Clin. Chem. 61, 714–723. 10.1373/clinchem.2014.232918 25779987

[B98] Pacchiarotta T. Hensbergen P. J. Wuhrer M. van Nieuwkoop C. Nevedomskaya E. Derks R. J. (2012). Fibrinogen alpha chain O-glycopeptides as possible markers of urinary tract infection. J. Proteomics 75, 1067–1073. 10.1016/j.jprot.2011.10.021 22075168

[B99] Palomino T. V. Sheridan A. Muddiman D. C. Brown A. C. (2024). In-depth characterization of *N*-glycosylation and sialic acid content in fetal and adult fibrinogen. Res. Pract. Thromb. Haemost. 8, 102618. 10.1016/j.rpth.2024.102618 39668886 PMC11635000

[B100] Pandey V. K. Sharma R. Prajapati G. K. Mohanta T. K. Mishra A. K. (2022). N-glycosylation, a leading role in viral infection and immunity development. Mol. Biol. Rep. 49, 8109–8120. 10.1007/s11033-022-07359-4 35364718 PMC8974804

[B101] Perween S. Abidi M. Faizy A. F. Moinuddin (2019). Post-translational modifications on glycated plasma fibrinogen: a physicochemical insight. Int. J. Biol. Macromol. 126, 1201–1212. 10.1016/j.ijbiomac.2019.01.018 30625358

[B102] Perween S. Abidi M. Faiz Faizy A. Moinuddin (2022). Biophysical changes in methylglyoxal modified fibrinogen and its role in the immunopathology of type 2 diabetes mellitus. Int. J. Biol. Macromol. 202, 199–214. 10.1016/j.ijbiomac.2021.12.161 34999047

[B103] Pieters M. Covic N. Loots d.T. van der Westhuizen F. H. van Zyl D. G. Rheeder P. (2006). The effect of glycaemic control on fibrin network structure of type 2 diabetic subjects. Thromb. Haemost. 96, 623–629. 10.1160/th06-07-0390 17080220

[B104] Pieters M. van Zyl D. G. Rheeder P. Jerling J. C. Loots d.T. van der Westhuizen F. H. (2007). Glycation of fibrinogen in uncontrolled diabetic patients and the effects of glycaemic control on fibrinogen glycation. Thromb. Res. 120, 439–446. 10.1016/j.thromres.2006.10.016 17156827

[B105] Pieters M. Covic N. van der Westhuizen F. H. Nagaswami C. Baras Y. Toit Loots D. (2008). Glycaemic control improves fibrin network characteristics in type 2 diabetes - a purified fibrinogen model. Thromb. Haemost. 99, 691–700. 10.1160/TH07-11-0699 18392327 PMC2854507

[B106] Pu Q. Yu C. (2014). Glycosyltransferases, glycosylation and atherosclerosis. Glycoconj J. 31, 605–611. 10.1007/s10719-014-9560-8 25294497

[B107] Radovani B. Vučković F. Maggioni A. P. Ferrannini E. Lauc G. Gudelj I. (2023). IgG N-glycosylation is altered in coronary artery disease. Biomolecules 13, 375. 10.3390/biom13020375 36830744 PMC9953309

[B108] Rehman S. Faisal M. Alatar A. A. Ahmad S. (2020). Physico-chemical changes induced in the serum proteins immunoglobulin G and fibrinogen mediated by methylglyoxal. Curr. Protein Pept. Sci. 21, 916–923. 10.2174/1389203720666190618095719 31244422

[B109] Rehman S. Song J. Faisal M. Alatar A. A. Akhter F. Ahmad S. (2021a). The neoepitopes on methylglyoxal- (MG-) glycated fibrinogen generate autoimmune response: its role in diabetes, atherosclerosis, and diabetic atherosclerosis subjects. Oxid. Med. Cell Longev. 2021, 6621568. 10.1155/2021/6621568 34970417 PMC8714332

[B110] Rehman S. Alouffi S. Faisal M. Qahtan A. A. Alatar A. A. Ahmad S. (2021b). Methylglyoxal mediated glycation leads to neo-epitopes generation in fibrinogen: role in the induction of adaptive immune response. Int. J. Biol. Macromol. 175, 535–543. 10.1016/j.ijbiomac.2021.01.197 33529635

[B111] Ridgway H. J. Brennan S. O. Loreth R. M. George P. M. (1997). Fibrinogen Kaiserslautern (gamma 380 Lys to Asn): a new glycosylated fibrinogen variant with delayed polymerization. Br. J. Haematol. 99, 562–569. 10.1046/j.1365-2141.1997.4363246.x 9401066

[B112] Riggs K. A. Joshi P. H. Khera A. Singh K. Akinmolayemi O. Ayers C. R. (2019). Impaired HDL metabolism links GlycA, A novel inflammatory marker, with incident cardiovascular events. J. Clin. Med. 8, 2137. 10.3390/jcm8122137 31817053 PMC6947609

[B113] Riggs K. A. Joshi P. H. Khera A. Otvos J. D. Greenland P. Ayers C. R. (2022). GlycA, hsCRP differentially associated with MI, ischemic stroke: in the Dallas heart study and multi-ethnic study of atherosclerosis: GlycA, hsCRP differentially associated MI, stroke. Am. J. Prev. Cardiol. 12, 100373. 10.1016/j.ajpc.2022.100373 36061365 PMC9428838

[B114] Robinson P. W. Jury D. R. Langdon A. G. (1991). The relative clotting activity of glycated and non-glycated forms of fibrinogen. Ann. Clin. Biochem. 28 (Pt 6), 618–619. 10.1177/000456329102800613 1776813

[B115] Rooney M. R. Daya N. Tang O. McEvoy J. W. Coresh J. Christenson R. H. (2022). Glycated albumin and risk of mortality in the US adult population. Clin. Chem. 68, 422–430. 10.1093/clinchem/hvab232 35092265 PMC8897960

[B116] Rudd P. M. Elliott T. Cresswell P. Wilson I. A. Dwek R. A. (2001). Glycosylation and the immune system. Science 291, 2370–2376. 10.1126/science.291.5512.2370 11269318

[B117] Schjoldager K. T. Narimatsu Y. Joshi H. J. Clausen H. (2020). Global view of human protein glycosylation pathways and functions. Nat. Rev. Mol. Cell Biol. 21, 729–749. 10.1038/s41580-020-00294-x 33087899

[B118] Shamsi A. Amani S. Alam M. T. Naeem A. (2016). Aggregation as a consequence of glycation: insight into the pathogenesis of arthritis. Eur. Biophys. J. 45, 523–534. 10.1007/s00249-016-1119-0 27017355

[B119] Shen C. Y. Lu C. H. Wu C. H. Li K. J. Kuo Y. M. Hsieh S. C. (2020). The development of maillard reaction, and advanced glycation end product (AGE)-Receptor for AGE (RAGE) signaling inhibitors as novel therapeutic strategies for patients with AGE-related diseases. Molecules 25, 5591. 10.3390/molecules25235591 33261212 PMC7729569

[B120] Shin A. Connolly S. Kabytaev K. (2023). Protein glycation in diabetes mellitus. Adv. Clin. Chem. 113, 101–156. 10.1016/bs.acc.2022.11.003 36858645

[B121] Siddiqui Z. Faisal M. Alatar A. A. Ahmad S. (2019). Glycation of hemoglobin leads to the immunogenicity as a result of neo-epitope generation. Int. J. Biol. Macromol. 123, 427–435. 10.1016/j.ijbiomac.2018.11.063 30445080

[B122] Šoić D. Kifer D. Szavits-Nossan J. Blivajs A. Đerek L. Rudan D. (2025). High-throughput site-specific N-glycosylation profiling of human fibrinogen in atrial fibrillation. J. Proteome Res. 24, 2121–2134. 10.1021/acs.jproteome.5c00096 40099449 PMC11976851

[B123] Spiro R. G. (2002). Protein glycosylation: nature, distribution, enzymatic formation, and disease implications of glycopeptide bonds. Glycobiology 12, 43R–56R. 10.1093/glycob/12.4.43r 12042244

[B124] Sugo T. Nakamikawa C. Takano H. Mimuro J. Yamaguchi S. Mosesson M. W. (1999). Fibrinogen Niigata with impaired fibrin assembly: an inherited dysfibrinogen with a Bβ asn-160 to ser substitution associated with extra glycosylation at Bβ Asn-158. Blood 94, 3806–3813. 10.1182/blood.v94.11.3806.423a17_3806_3813 10572095

[B125] Svensson J. Bergman A. C. Adamson U. Blombäck M. Wallén H. Jörneskog G. (2012). Acetylation and glycation of fibrinogen *in vitro* occur at specific lysine residues in a concentration dependent manner: a mass spectrometric and isotope labeling study. Biochem. Biophys. Res. Commun. 421, 335–342. 10.1016/j.bbrc.2012.03.154 22507986

[B126] Taniguchi N. Takahashi M. Kizuka Y. Kitazume S. Shuvaev V. V. Ookawara T. (2016). Glycation vs. glycosylation: a tale of two different chemistries and biology in Alzheimer's disease. Glycoconj J. 33, 487–497. 10.1007/s10719-016-9690-2 27325408

[B127] Todoriki S. Hosoda Y. Yamamoto T. Watanabe M. Sekimoto A. Sato H. (2022). Methylglyoxal induces inflammation, metabolic modulation and oxidative stress in myoblast cells. Toxins (Basel) 14, 263. 10.3390/toxins14040263 35448872 PMC9030564

[B128] Uceda A. B. Mariño L. Casasnovas R. Adrover M. (2024). An overview on glycation: molecular mechanisms, impact on proteins, pathogenesis, and inhibition. Biophys. Rev. 16, 189–218. 10.1007/s12551-024-01188-4 38737201 PMC11078917

[B129] Vadseth C. Souza J. M. Thomson L. Seagraves A. Nagaswami C. Scheiner T. (2004). Pro-thrombotic state induced by post-translational modification of fibrinogen by reactive nitrogen species. J. Biol. Chem. 279, 8820–8826. 10.1074/jbc.M306101200 14681238

[B130] Vistoli G. De Maddis D. Cipak A. Zarkovic N. Carini M. Aldini G. (2013). Advanced glycoxidation and lipoxidation end products (AGEs and ALEs): an overview of their mechanisms of formation. Free Radic. Res. 47 (Suppl. 1), 3–27. 10.3109/10715762.2013.815348 23767955

[B131] Vu D. Neerman-Arbez M. (2007). Molecular mechanisms accounting for fibrinogen deficiency: from large deletions to intracellular retention of misfolded proteins. J. Thromb. Haemost. 5 (Suppl. 1), 125–131. 10.1111/j.1538-7836.2007.02465.x 17635718

[B132] Wang G. Wang Y. Yang Q. Xu C. Zheng Y. Wang L. (2022). Metformin prevents methylglyoxal-induced apoptosis by suppressing oxidative stress *in vitro* and *in vivo* . Cell Death Dis. 13, 29. 10.1038/s41419-021-04478-x 35013107 PMC8748764

[B133] Wang Y. Liu H. Hu X. Wang A. Kang S. Zhang L. (2023). Association between hemoglobin glycation index and 5-year major adverse cardiovascular events: the REACTION cohort study. Chin. Med. J. Engl. 136, 2468–2475. 10.1097/CM9.0000000000002717 37265382 PMC10586840

[B134] Wang Y. Chen J. Zheng Y. Jiang J. Wang L. Wu J. (2024). Glucose metabolite methylglyoxal induces vascular endothelial cell pyroptosis via NLRP3 inflammasome activation and oxidative stress *in vitro* and *in vivo* . Cell Mol. Life Sci. 81, 401. 10.1007/s00018-024-05432-8 39269632 PMC11399538

[B135] Wattchow N. E. Pullen B. J. Indraratna A. D. Nankivell V. Everest-Dass A. Psaltis P. J. (2025). The emerging role of glycans and the importance of sialylation in cardiovascular disease. Atherosclerosis 403, 119172. 10.1016/j.atherosclerosis.2025.119172 40138819

[B136] Weisel J. W. (2005). Fibrinogen and fibrin. Adv. Protein Chem. 70, 247–299. 10.1016/S0065-3233(05)70008-5 15837518

[B137] Weisel J. W. Litvinov R. I. (2017). Fibrin Formation, structure and properties. Subcell. Biochem. 82, 405–456. 10.1007/978-3-319-49674-0_13 28101869 PMC5536120

[B138] Whittaker A. Dinu M. Cesari F. Gori A. M. Fiorillo C. Becatti M. (2017). A khorasan wheat-based replacement diet improves risk profile of patients with type 2 diabetes mellitus (T2DM): a randomized crossover trial. Eur. J. Nutr. 56, 1191–1200. 10.1007/s00394-016-1168-2 26853601 PMC5346426

[B139] Wolberg A. S. (2023). Fibrinogen and fibrin: synthesis, structure, and function in health and disease. J. Thromb. Haemost. 21, 3005–3015. 10.1016/j.jtha.2023.08.014 37625698 PMC10592048

[B140] Woodhead J. L. Nagaswami C. Matsuda M. Arocha-Piñango C. L. Weisel J. W. (1996). The ultrastructure of fibrinogen Caracas II molecules, fibers, and clots. J. Biol. Chem. 271, 4946–4953. 10.1074/jbc.271.9.4946 8617768

[B141] Yamazumi K. Shimura K. Terukina S. Takahashi N. Matsuda M. (1989). A gamma methionine-310 to threonine substitution and consequent N-glycosylation at gamma asparagine-308 identified in a congenital dysfibrinogenemia associated with posttraumatic bleeding, fibrinogen Asahi. J. Clin. Invest 83, 1590–1597. 10.1172/JCI114056 2496144 PMC303865

[B142] Yubero-Serrano E. M. Pérez-Martínez P. (2020). Advanced glycation end products and their involvement in cardiovascular disease. Angiology 71, 698–700. 10.1177/0003319720916301 32242451

[B143] Zauner G. Hoffmann M. Rapp E. Koeleman C. A. Dragan I. Deelder A. M. (2012). Glycoproteomic analysis of human fibrinogen reveals novel regions of O-glycosylation. J. Proteome Res. 11, 5804–5814. 10.1021/pr3005937 23050552

[B144] Zhang J. Liu Y. Deng X. Chen L. Yang X. Yu C. (2018). ST6GAL1 negatively regulates monocyte transendothelial migration and atherosclerosis development. Biochem. Biophys. Res. Commun. 500, 249–255. 10.1016/j.bbrc.2018.04.053 29654763

[B145] Zhao H. Hu Q. Chen J. Ling Q. Yan Z. Yu P. (2023). Glycated albumin and risk of cardiovascular diseases and mortality in patients with and without dialysis: a meta-analysis. Diabetes Obes. Metab. 25, 2203–2217. 10.1111/dom.15097 37132338

